# Comparative Transcriptome Analysis Reveals Different Molecular Mechanisms of *Bacillus coagulans* 2-6 Response to Sodium Lactate and Calcium Lactate during Lactic Acid Production

**DOI:** 10.1371/journal.pone.0124316

**Published:** 2015-04-15

**Authors:** Jiayang Qin, Xiuwen Wang, Landong Wang, Beibei Zhu, Xiaohua Zhang, Qingshou Yao, Ping Xu

**Affiliations:** 1 College of Pharmacy, Binzhou Medical University, Yantai, People's Republic of China; 2 State Key Laboratory of Microbial Metabolism, and School of Life Sciences & Biotechnology, Shanghai Jiao Tong University, Shanghai, People’s Republic of China; Public Health Research Institute at RBHS, UNITED STATES

## Abstract

Lactate production is enhanced by adding calcium carbonate or sodium hydroxide during fermentation. However, *Bacillus coagulans* 2-6 can produce more than 180 g/L L-lactic acid when calcium lactate is accumulated, but less than 120 g/L L-lactic acid when sodium lactate is formed. The molecular mechanisms by which *B*. *coagulans* responds to calcium lactate and sodium lactate remain unclear. In this study, comparative transcriptomic methods based on high-throughput RNA sequencing were applied to study gene expression changes in *B*. *coagulans* 2-6 cultured in non-stress, sodium lactate stress and calcium lactate stress conditions. Gene expression profiling identified 712 and 1213 significantly regulated genes in response to calcium lactate stress and sodium lactate stress, respectively. Gene ontology assignments of the differentially expressed genes were performed. KEGG pathway enrichment analysis revealed that ‘ATP-binding cassette transporters’ were significantly affected by calcium lactate stress, and ‘amino sugar and nucleotide sugar metabolism’ was significantly affected by sodium lactate stress. It was also found that lactate fermentation was less affected by calcium lactate stress than by sodium lactate stress. Sodium lactate stress had negative effect on the expression of ‘glycolysis/gluconeogenesis’ genes but positive effect on the expression of ‘citrate cycle (TCA cycle)’ genes. However, calcium lactate stress had positive influence on the expression of ‘glycolysis/gluconeogenesis’ genes and had minor influence on ‘citrate cycle (TCA cycle)’ genes. Thus, our findings offer new insights into the responses of *B*. *coagulans* to different lactate stresses. Notably, our RNA-seq dataset constitute a robust database for investigating the functions of genes induced by lactate stress in the future and identify potential targets for genetic engineering to further improve L-lactic acid production by *B*. *coagulans*.

## Introduction

Lactic acid has wide applications in the industries of food, medicine, brewing, leather, textiles, environmental protection and agriculture [1–3]. The major industrial application of lactic acid is the production of polylactic acid, a biodegradable polymer. Given the biocompatibility, biodegradability and excellent mechanical properties of polylactic acid, it is considered the most promising novel packaging material of this century. It can replace non-degradable petrochemical materials such as polyethylene, polypropylene and polystyrene. Importantly, widespread use of polylactic acid has the potential to solve ‘white pollution’ and mitigate the current energy crisis [2, 4].

During fermentative production of lactic acid, a neutraliser such as calcium carbonate or sodium hydroxide is required to maintain neutral or mildly acidic conditions for the fermentation liquid. Sodium lactate, calcium lactate or another lactate can accumulate in the fermentation broth depending on the type of neutraliser selected. High concentrations of lactate result in stress effects on lactic acid producers, and are among the key factors limiting the enhancement of lactic acid yields [5]. Previous studies indicated that the maximum yield of lactic acid can exceed 200 g/L when calcium lactate accumulates in the fermentation broth [6, 7]; however, the lactic acid yield is generally less than 125 g/L when sodium lactate is formed [5, 8]. Therefore, the influences of lactate on lactic acid production may be different depending on the type of lactic acid formed.

*Bacillus coagulans* strains are L-lactic acid producers with industrial potential because they can tolerate high fermentation temperatures, their culture media do not need sterilisation and their products exhibit high optical purity [9, 10]. *B*. *coagulans* 2–6 can produce more than 180 g/L L-lactic acid with high optical purity when calcium lactate is accumulated, but less than 120 g/L L-lactic acid when sodium lactate is formed [5, 11]. Under the same concentration of lactates, sodium lactate results in stronger stress on *B*. *coagulans* 2–6 compared with calcium lactate [12]. However, the mechanisms by which *B*. *coagulans* strains respond to different lactates remain unclear.

Omics tools are effective for studying the response mechanisms of bacteria to stress conditions. Wang et al. [12] reported a comparative proteomic analysis of *B*. *coagulans* 2–6 in response to lactate stress and identified four highly expressed proteins under calcium lactate stress. In the present study, we conducted comparative transcriptomics to study differential expression of genes among *B*. *coagulans* 2–6 cultured in normal, sodium lactate stress and calcium lactate stress conditions. Similarities and differences in the response of *B*. *coagulans* 2–6 to sodium lactate and calcium lactate were also investigated.

## Materials and Methods

### Bacterial strains and cultivation conditions

The *B*. *coagulans* wild-type strain 2–6 [11, 13] was used in this study. It was cultivated at 50°C with shaking at 150 rpm in non-stress medium (GY medium: 40 g/L glucose, 10 g/L yeast extract, 1 g/L CaCl_2_, pH 6.7) until the cell dry weight (CDW) reached 2.0. Then 5 ml bacterial liquid was inoculated into 100 ml fresh non-stress medium and lactate stress medium (GY medium with 0.5 M sodium lactate or 0.25 M calcium lactate), and incubated at 50°C with shaking at 150 rpm until CDW reached 0.4 g/L at late exponential stage of the cell growth as reported before [12]. Cells were harvested for preparation of total RNA.

### Library preparation and RNA sequencing (RNA-seq)

Total RNA of *B*. *coagulans* 2–6 was isolated using TRIzol reagent according to the manufacturer’s protocol (Invitrogen, Carlsbad, CA, USA) and then treated with DNase I. The quality and quantity of purified RNA were determined by measuring the absorbance at 260 nm/280 nm (A260/A280) using a Nanodrop ND-1000 spectrophotometer (LabTech, Wilmington, MA, USA). RNA integrity was further verified by electrophoresis through 1.5% (w/v) agarose gel. mRNA was enriched from total RNA using a Ribo-Zero Magnetic Kit (Gram-Positive Bacteria) (EpiCentre, Madison, WY, USA). Synthesis of double-stranded cDNA from the extracted mRNA was done using the Truseq RNA sample prep kit (Illumina, San Diego, CA, USA), followed by 15 cycles of enrichment PCR. Sequencing was carried out on the Illumina HiSeq 2000 platform (Majorbio Bio-Pharm Technology Co., Ltd., Shanghai, China). All sequencing data have been deposited in the NCBI Sequence Read Archive under accession numbers SRX700697 (GY), SRX700698 (CA) and SRX 700710 (NA).

### Transcriptome analysis

The raw data were initially processed to obtain clean reads by removing the adapter sequences and low quality bases. The clean reads were then aligned to the reference genome sequence of *B*. *coagulans* 2–6 (accession number: NC_015634) using the short sequence alignment software Bowtie 2 [14]. To identify differentially expressed genes (DEGs) between the two different treatment conditions, the expression level for each transcript was calculated by RSEM (http://deweylab.biostat.wisc.edu/rsem/) using the fragments per kilobase of exon per million mapped reads method [15]. The R statistical package software EdgeR (http://www.bioconductor.org/packages/2.12/bioc/html/edgeR.html) was employed to quantify differential gene expression [16]. Genes were defined as differentially expressed if they exhibited two-fold or greater change between the samples under a false discovery rate (FDR) of 5% or less. In addition, functional enrichment analyses, including gene ontology (GO) and Kyoto Encyclopaedia of Genes and Genomes (KEGG), were performed to identify which DEGs were significantly enriched in GO terms and metabolic pathways compared with the whole-transcriptome background using the formula described in previous studies [17, 18]. A Bonferroni-corrected P-value ≤ 0.05 was considered statistically significant.

### Quantitative real-time PCR (qRT-PCR) verification

Five genes belonging to 5 different operons were chosen for confirmation of RNA-seq data by qRT-PCR. Total RNA was extracted using the EasyPure RNA Purification Kit (TRANSGEN BIOTECH, Beijing, China). Then, the total RNA sample was treated with DNase and qRT-PCR was performed on RNA samples to confirm the absence of DNA contamination using 16S primers [19]. qRT-PCR was performed using the TransScript II Green One-Step qRT-PCR SuperMix (TRANSGEN BIOTECH, Beijing, China) and the Corbett Rotor Gene 3000 (Corbett Robotics, Sydney, Australia) according to the manufacturer’s instructions. The genes and primers used for qRT-PCR are shown in S1 Table. The relative gene expression data were analyzed using the 2^-ΔΔCt^ method as described by Tan et al. [19]. Rotor-Gene Real-Time Analysis Software 6.0 was used for data analysis (Corbett Robotics, Sydney, Australia). All qRT-PCR runs were conducted with three biological and three technical replicates.

## Results

### Illumina draft reads

Large volumes of data were generated with Illumina HiSeq 2000 sequencing of the three *B*. *coagulans* cDNA samples, namely, GY (untreated control), CA, (calcium lactate-stressed sample) and NA (sodium lactate-stressed sample). Each cDNA fragment yielded two 101 base pair long paired-end (PE) reads. After removing low quality reads and trimming off adapter sequences, 13,747,126 (GY), 13,464,898 (CA) and 14,974,752 (NA) high-quality, clean PE sequencing reads with a total of 1,334,784,442 (GY), 1,306,406,094 (CA) and 1,456,148,628 (NA) nucleotides were obtained for the three samples. Clean reads were aligned to the reference genome. The mapping rates were 97.9% (GY), 95.9% (CA) and 95.6% (NA).

### Gene ontology (GO) functional annotation of transcripts

**Fig 1:**
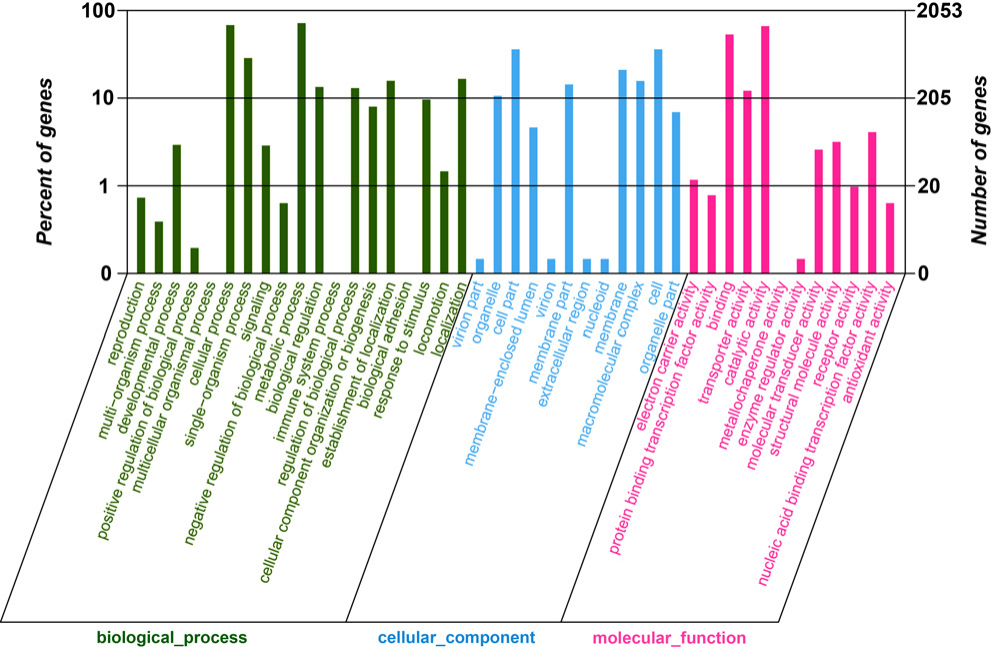
Gene Ontology (GO) functional annotation of transcripts. All 2,053 transcripts were assigned to at least one GO term and were grouped into three main GO categories and 43 groups, 19 groups in biological process, 12 in cellular component, and 12 in molecular function. The right-hand Y-axis represents the number of genes in a sub-category. The left-hand Y-axis indicates the percentage of a specific sub-category of genes in each main category.

GO assignments were used to classify the functions of the *B*. *coagulans* genes under lactate stress. Based on sequence homology, 2053 transcript sequences were assigned at least one GO term, including 43 functional groups at the second level (Fig 1). Among these groups, ‘catalytic activity’ (1359 sequences, 66.2%), ‘binding’ (1091 sequences, 53.1%) and ‘single-organism process’ (587 sequences, 28.6%) were the most abundant subcategories for ‘molecular function’. ‘Cell’ (739 sequences, 36.0%), ‘cell part’ (739 sequences, 36.0%) and ‘membrane’ (431 sequences, 21.0%) terms were dominant in ‘cellular component’. ‘Metabolic process’ (1472 sequences, 71.7%), ‘cellular process’ (1396 sequences, 68.0%) and ‘single-organism process’ (587 sequences, 28.6%) were represented the highest under ‘biological process’.

### Differentially expressed genes (DEGs) involved in response to lactate stress in *B*. *coagulans*

**Fig 2:**
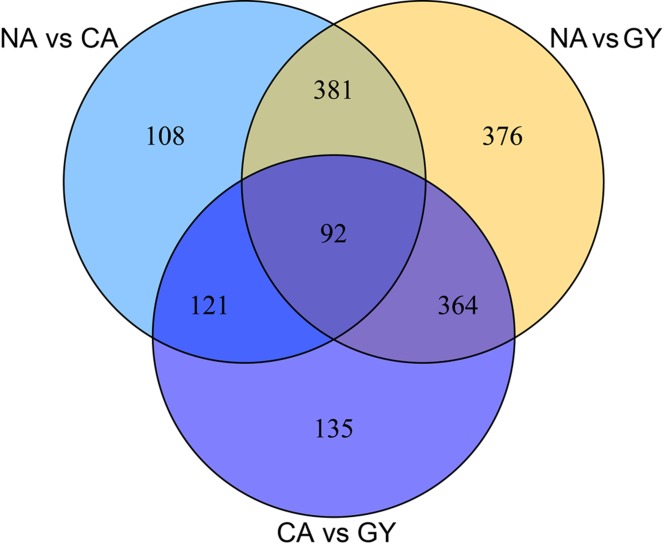
Differentially expressed genes that are unique or shared among three groups of GY, NA and CA. NA vs GY refers to the comparison between sodium lactate stressed (NA) and untreated (GY) groups. CA vs GY refers to the comparison between calcium lactate stressed (CA) and GY groups. NA vs CA refers to the comparison between NA and CA groups. Numbers in each section of the figure indicate the numbers of differently expressed genes in the indicated comparison.

Comparison of gene expression showed that a total of 1577 unigenes were differentially expressed between any two-way comparison of GY, CA and NA (fold changes ≥2 or ≤−2; FDR<0.05), including 712 isogenes between GY and CA (GY vs CA), 702 isogenes between NA and CA (NA vs CA) and 1213 isogenes between NA and GY (NA vs GY). The amounts of significantly up- and down-regulated genes in NA were 543 and 670, respectively, compared with those in GY. The amounts of significantly up- and down-regulated genes in CA were 398 and 314, respectively, compared with those in GY. Moreover, 364 unigenes were significantly differentially expressed in both CA vs GY and NA vs GY; 121 unigenes were significantly differentially expressed in both CA vs GY and NA vs CA; and 381 unigenes were significantly differentially expressed in both NA vs GY and NA vs CA. Only 92 unigenes were significantly differentially expressed in CA vs GY, NA vs GY and NA vs CA. The numbers of all differentially expressed genes among the three groups of GY, CA and NA are illustrated in Fig 2.

### GO assignments of DEGs

GO assignments of DEGs in response to calcium lactate stress and sodium lactate stress were performed. The results are shown in Table 1. The 398 significantly upregulated genes in CA compared with those in GY were assigned 34 GO terms, namely, 14 subcategories of biological process, 9 subcategories of cellular component and 11 subcategories of molecular function. The 314 significantly down-regulated genes in CA compared with those in GY were assigned 34 GO terms, namely, 15 subcategories of biological process, 10 subcategories of cellular component and 9 subcategories of molecular function. The 543 significantly upregulated genes in NA compared with those in GY were assigned 39 GO terms, namely, 17 subcategories of biological process, 12 subcategories of cellular component and 10 subcategories of molecular function. The 670 significantly down-regulated genes in NA compared with those in GY were assigned 40 GO terms, namely, 18 subcategories of biological process, 10 subcategories of cellular component and 12 subcategories of molecular function. The significantly up- or down-regulated genes in the corresponding GO terms are listed in Table 1, S1 Data and S2 Data. Taken together, the results indicate differences in gene expression changes in response to calcium lactate stress and sodium lactate stress in *B*. *coagulans* 2–6.

**Table 1:** Gene ontology (GO) assignments of differentially expressed genes.

GO type	GO term	GO id	CA vs GY		NA vs GY	
			up	down	up	down
Biological process	biological regulation	0065007	39*	24	63	61
	cellular component organization or biogenesis	0071840	8	8	25	40
	cellular process	0009987	161	145	237	359
	developmental process	0032502	3	2	14	18
	establishment of localization	0051234	30	69	52	105
	immune system process	0002376	0	1	0	1
	localization	0051179	30	71	52	117
	locomotion	0040011	1	4	2	19
	metabolic process	0008152	195	149	258	368
	multi-organism process	0051704	0	2	1	3
	negative regulation of biological process	0048519	2	2	4	3
	positive regulation of biological process	0048518			1	1
	regulation of biological process	0050789	37	23	62	60
	response to stimulus	0050896	34	23	35	50
	Signaling	0023052	3	6	7	16
	single-organism process	0044699	153	133	201	311
	biological adhesion	0022610	1	0	1	1
	reproduction	0000003			3	1
Cellular component	Cell	0005623	79	83	129	201
	cell part	0044464	79	83	129	201
	extracellular region	0005576	1	3	1	2
	extracellular region part	0044421	0	1		
	macromolecular complex	0032991	34	29	58	89
	Membrane	0016020	36	78	65	131
	membrane-enclosed lumen	0031974	1	2	2	3
	membrane part	0044425	22	56	46	89
	Nucleoid	0009295			1	1
	Organelle	0043226	6	10	13	51
	organelle part	0044422	2	5	7	26
	Virion	0019012			2	0
	virion part	0044423			2	0
Molecular function	Binding	0005488	145	123	213	266
	catalytic activity	0003824	185	143	236	340
	electron carrier activity	0009055	8	6	7	2
	metallochaperone activity	0016530	0	1	0	1
	molecular transducer activity	0060089	3	5	5	14
	nucleic acid binding transcription factor activity	0001071	12	7	26	15
	receptor activity	0004872	1	2	1	6
	structural molecule activity	0005198	4	5	3	31
	transporter activity	0005215	20	55	35	83
	antioxidant activity	0016209	5	0	2	1
	enzyme regulator activity	0030234	2	0	0	1
	protein binding transcription factor activity	0000988	3	0	4	3

*These are numbers of significantly up- or down-regulated genes in the corresponding GO term

### GO and pathway functional enrichment analysis of DEGs

GO function and pathway enrichment analyses were performed on all DEGs. According to GO functional enrichment analysis between CA and GY, the GO term ‘response to stress’ was represented the most and included 96 unigenes and 41 significantly differentially expressed unigenes. Analysis between NA and GY revealed one significantly enriched GO term. This GO term was ‘biological process’ (GO: 0008150), which involved 1813 unigenes and 793 significantly differentially expressed unigenes. No significantly enriched GO term was found between NA and CA.

Kyoto Encyclopaedia of Genes and Genomes (KEGG) enrichment analysis showed that the DEGs between the CA and GY groups were significantly involved in the ‘ATP-binding cassette (ABC) transporters’ (KEGG: ko02010) pathway, which included 96 unigenes and 37 significantly differentially expressed unigenes. The DEGs between the NA and GY groups were significantly involved in the ‘amino sugar and nucleotide sugar metabolism’ pathway, which included 35 unigenes and 26 significantly differentially expressed unigenes. The DEGs between the NA and CA groups were significantly involved in the ‘amino sugar and nucleotide sugar metabolism’ (KEGG: ko00520) pathway, which included 35 unigenes and 22 significantly differentially expressed unigenes.

### Identification and comparison of DEGs involved in ‘ABC transporters’

‘ABC transporters’, which are also called traffic ATPases, use the binding and hydrolysis of ATP to power the translocation of a diverse assortment of substrates across biological membranes, ranging from ions to macromolecules [20]. ‘ABC transporters’ either import or export substrates with relative specificity for a given substrate. Dozens of ABC transporters were identified by genome analysis in *Escherichia coli* [21] and *Bacillus subtilis* [22].

KEGG pathway enrichment analysis revealed that ‘ABC transporters’ was significantly affected by calcium lactate stress. A total of 10 significantly upregulated genes (Table 2) and 27 significantly down-regulated genes (S2 Table) were involved in ‘ABC transporters’ under calcium lactate stress condition (CA group), compared with those under non-stress condition (GY group). Fourteen significantly upregulated genes (Table 3) and 35 significantly down-regulated genes (S3 Table) were involved in ‘ABC transporters’ under sodium lactate stress condition (NA group) compared with those under non-stress condition (GY group). Six genes, namely, glycine betaine/L-proline ABC transporter ATPase (BCO26_0425), binding-protein-dependent transport system inner membrane protein (BCO26_0553), NMT1/THI5 like domain-containing protein (BCO26_0554), ABC transporter-like protein (BCO26_0555), periplasmic solute binding protein (BCO26_2084) and ABC transporter (BCO26_2523), were significantly upregulated in both the CA and NA groups compared with those in the GY group. Four genes, namely, phosphate binding protein (BCO26_0705), phosphate ABC transporter permease (BCO26_0706, BCO26_0707) and ABC transporter-like protein (BCO26_0743), were significantly up-regulated in CA but not in NA compared with GY. Eight genes, namely binding-protein-dependent transport system inner membrane protein (BCO26_0422), glycine betaine ABC transporter substrate-binding protein (BCO26_0423), binding-protein-dependent transport system inner membrane protein (BCO26_0424), transport system permease (BCO26_0685, BCO26_0686), ABC transporter-like protein (BCO26_0687), periplasmic binding protein (BCO26_0688) and ABC transporter-like protein (BCO26_2588), were significantly upregulated in NA but not in CA compared with GY. The significantly down-regulated genes involved in ‘ABC transporters’ under the two kinds of lactate stress were mostly the same (S2 Table and S3 Table). However, sodium lactate induced more significantly down-regulated genes than calcium lactate (35 to 27) and higher average down-regulation (fold change ranging from 3.72 to 3.06).

**Table 2:** Significantly upregulated genes involved in ‘ABC transporters’ under calcium lactate stress.

Gene ID	Description	FDR	Fold change
BCO26_0425	glycine betaine/L-proline ABC transporter ATPase	6.01E-03	2.30
BCO26_0553	binding-protein-dependent transport system inner membrane protein	3.04E-03	2.55
BCO26_0554	NMT1/THI5 like domain-containing protein	1.80E-02	2.03
BCO26_0555	ABC transporter-like protein	2.38E-02	1.94
BCO26_0705	phosphate binding protein	2.62E-06	4.16
BCO26_0706	phosphate ABC transporter permease	8.04E-04	2.98
BCO26_0707	phosphate ABC transporter permease	6.70E-03	2.40
BCO26_0743	ABC transporter-like protein	5.19E-03	2.36
BCO26_2084	periplasmic solute binding protein	1.52E-02	2.05
BCO26_2523	ABC transporter	9.44E-03	2.25

**Table 3:** Significantly upregulated genes involved in ‘ABC transporters’ under sodium lactate stress.

Gene ID	Description	FDR	Fold change
BCO26_0422	binding-protein-dependent transport system inner membrane protein	1.00E-02	2.02
BCO26_0423	glycine betaine ABC transporter substrate-binding protein	4.04E-02	1.60
BCO26_0424	binding-protein-dependent transport system inner membrane protein	4.16E-02	1.60
BCO26_0425	glycine betaine/L-proline ABC transporter ATPase	1.12E-02	1.98
BCO26_0553	binding-protein-dependent transport system inner membrane protein	1.15E-02	2.04
BCO26_0554	NMT1/THI5 like domain-containing protein	5.03E-03	2.22
BCO26_0555	ABC transporter-like protein	3.86E-04	2.82
BCO26_0685	transport system permease	5.90E-05	3.20
BCO26_0686	transport system permease	3.95E-04	2.81
BCO26_0687	ABC transporter-like protein	2.20E-03	2.42
BCO26_0688	periplasmic binding protein	1.03E-03	2.59
BCO26_2084	periplasmic solute binding protein	4.48E-02	1.57
BCO26_2523	ABC transporter	6.40E-03	2.21
BCO26_2588	ABC transporter-like protein	4.57E-02	1.94

### Identification and comparison of DEGs involved in ‘amino sugar and nucleotide sugar metabolism’

In nucleotide sugar metabolism, nucleotide sugars act as donors for sugar residues in the glycosylation reactions that produce polysaccharides, which are important constituents of the cell wall [23]. Nucleotide sugars are also intermediates in nucleotide sugar interconversions, which produce some of the activated sugars necessary for glycosylation reactions [24]. KEGG pathway enrichment analysis revealed that ‘amino sugar and nucleotide sugar metabolism’ was significantly affected by sodium lactate stress but not by calcium lactate stress. Fifteen significantly upregulated genes (Table 4) and 11 significantly down-regulated genes (S4 Table) were involved in ‘amino sugar and nucleotide sugar metabolism’ under sodium lactate stress condition (NA group) compared with those under non-stress condition (GY group). Eight significantly upregulated genes and one significantly down-regulated gene were involved in ‘amino sugar and nucleotide sugar metabolism’ under calcium lactate stress condition (CA group) compared with those under non-stress condition (GY group) (Table 5). Seven significantly regulated genes were found to exhibit the same trend under the two types of lactate stress conditions, namely, NAD(P)(+)-protein-arginine ADP-ribosyltransferase (BCO26_0186), glucosamine/fructose-6-phosphate aminotransferase (BCO26_0255), galactokinase (BCO26_0622), UDP-glucose 4-epimerase (BCO26_0623), N-acetylglucosamine-6-phosphate deacetylase (BCO26_0635), glucosamine-6-phosphate isomerise (BCO26_0636) and N-acylglucosamine-6-phosphate 2-epimerase (BCO26_2463). Eight genes were significantly upregulated specifically under sodium lactate stress, namely, UDP-N-acetylglucosamine pyrophosphorylase (BCO26_0036), phosphoglucosamine mutase (BCO26_0177), PTS system glucose subfamily transporter subunit IIA (BCO26_0204), phosphoglucomutase/phosphomannomutase alpha/beta/alpha domain I (BCO26_0507), galactose-1-phosphate uridylyltransferase (BCO26_0624), UDP-N-acetylenolpyruvoylglucosamine reductase (BCO26_1444), ROK family glucokinase (BCO26_1699) and UDP-N-acetylglucosamine 1-carboxyvinyltransferase (BCO26_2818). More genes were significantly down-regulated under sodium lactate stress than under calcium lactate stress (11 to 1). The four subunits of phosphoenolpyruvate-dependent sugar phosphotransferase system (PTS) mannose/fructose/sorbose family transporter (i.e., BCO26_0540, BCO26_0541, BCO26_0542 and BCO26_0543) were significantly down-regulated under sodium lactate stress (S4 Table), whereas the PTS system glucose subfamily transporter subunit IIA (BCO26_0204) was significantly upregulated (Table 4).

**Table 4:** Significantly upregulated genes involved in ‘amino sugar and nucleotide sugar metabolism’ under sodium lactate stress.

Gene ID	Description	FDR	Fold change
BCO26_0036	UDP-N-acetylglucosamine pyrophosphorylase	3.31E-03	2.29
BCO26_0177	phosphoglucosamine mutase	8.52E-03	2.04
BCO26_0186	NAD(P)(+)-protein-arginine ADP-ribosyltransferase	9.44E-07	4.03
BCO26_0204	PTS system glucose subfamily transporter subunit IIA	9.03E-03	2.16
BCO26_0255	glucosamine/fructose-6-phosphate aminotransferase	2.00E-09	5.17
BCO26_0507	phosphoglucomutase/phosphomannomutase alpha/beta/alpha domain I	3.58E-02	1.63
BCO26_0622	Galactokinase	1.44E-06	3.94
BCO26_0623	UDP-glucose 4-epimerase	3.77E-05	3.28
BCO26_0624	galactose-1-phosphate uridylyltransferase	1.80E-04	2.96
BCO26_0635	N-acetylglucosamine-6-phosphate deacetylase	1.84E-07	4.35
BCO26_0636	glucosamine-6-phosphate isomerase	5.23E-08	4.61
BCO26_1444	UDP-N-acetylenolpyruvoylglucosamine reductase	4.85E-02	1.54
BCO26_1699	ROK family glucokinase	4.25E-04	2.77
BCO26_2463	N-acylglucosamine-6-phosphate 2-epimerase	3.29E-09	5.08
BCO26_2818	UDP-N-acetylglucosamine 1-carboxyvinyltransferase	1.28E-03	2.51

**Table 5:** Significantly regulated genes involved in ‘amino sugar and nucleotide sugar metabolism’ under calcium lactate stress.

Gene ID	Description	FDR	Fold change
BCO26_0186	NAD(P)(+)-protein-arginine ADP-ribosyltransferase	3.19E-02	1.82
BCO26_0255	glucosamine/fructose-6-phosphate aminotransferase	2.02E-02	1.96
BCO26_0622	Galactokinase	1.68E-03	2.64
BCO26_0623	UDP-glucose 4-epimerase	4.24E-02	1.72
BCO26_0635	N-acetylglucosamine-6-phosphate deacetylase	5.19E-03	2.36
BCO26_0636	glucosamine-6-phosphate isomerase	4.01E-02	1.78
BCO26_2126	PTS system glucose subfamily transporter subunit IIA	7.25E-03	2.27
BCO26_2463	N-acylglucosamine-6-phosphate 2-epimerase	1.51E-02	2.06
BCO26_0354	nucleotide sugar dehydrogenase	2.71E-02	-1.87

### Identification and comparison of DEGs involved in ‘glycolysis/gluconeogenesis’

‘Glycolysis/gluconeogenesis’ is the key pathway for lactic acid production in *B*. *coagulans* 2–6. Therefore, we compared DEGs involved in ‘glycolysis/gluconeogenesis’ under lactate stress and non-stress conditions. Thirteen significantly upregulated genes (Table 6) and eight significantly down-regulated genes (S5 Table) were involved in ‘glycolysis/gluconeogenesis’ under sodium lactate stress condition (NA group) compared with those under non-stress condition (GY group). The pyruvate dehydrogenase complex, which mediates the conversion of pyruvic acid to acetyl-CoA, was significantly upregulated under sodium lactate stress compared with non-stress condition. The complex consists of pyruvate dehydrogenase components (BCO26_0962 and BCO26_0963), dihydrolipoamide transacetylase (BCO26_0964) and dihydrolipoamide dehydrogenase (BCO26_0965 and BCO26_1409).

**Table 6:** Significantly upregulated genes involved in ‘glycolysis/gluconeogenesis’ under sodium lactate stress.

Gene ID	Description	FDR	Fold change
BCO26_0204	PTS system glucose subfamily transporter subunit IIA	1.73E-03	2.90
BCO26_0492	glycoside hydrolase	1.22E-02	2.09
BCO26_0507	phosphoglucomutase/phosphomannomutase alpha/beta/alpha domain I	3.58E-02	1.63
BCO26_0625	aldose 1-epimerase	8.69E-03	2.19
BCO26_0962	pyruvate dehydrogenase E1 component subunit alpha	9.62E-04	2.76
BCO26_0963	transketolase central region	7.31E-04	2.85
BCO26_0964	hypothetical protein BCO26_0964	1.21E-03	2.70
BCO26_0965	dihydrolipoamide dehydrogenase	9.46E-04	2.76
BCO26_1409	dihydrolipoamide dehydrogenase	1.52E-02	2.30
BCO26_1699	ROK family glucokinase	4.72E-02	1.68
BCO26_1901	alcohol dehydrogenase GroES domain-containing protein	5.33E-04	2.95
BCO26_2235	enolase	1.68E-02	1.86
BCO26_2817	fructose-1,6-bisphosphatase	3.96E-03	2.24

Eleven significantly upregulated genes (Table 7) and five significantly down-regulated genes (S6 Table) were involved in ‘glycolysis/gluconeogenesis’ under calcium lactate stress condition (CA group) compared with those under non-stress condition (GY group) (Table 7). The key genes of the glycolysis pathway, including L-lactate dehydrogenase (BCO26_0531), pyruvate kinase (BCO26_1967), 6-phosphofructokinase (BCO26_1968), enolase (BCO26_2235), phosphoglycerate mutase (BCO26_2237), phosphoglycerate kinase (BCO26_2239) and fructose-bisphosphate aldolase (BCO26_2530), were all significantly upregulated (Table 7).

**Table 7:** Significantly upregulated genes involved in ‘glycolysis/gluconeogenesis’ under calcium lactate stress.

Gene ID	Description	FDR	Fold change
BCO26_0531	L-lactate dehydrogenase	3.35E-02	1.79
BCO26_1901	alcohol dehydrogenase GroES domain-containing protein	5.52E-03	2.41
BCO26_1967	pyruvate kinase	5.20E-03	2.33
BCO26_1968	6-phosphofructokinase	4.09E-04	2.96
BCO26_2126	PTS system glucose subfamily transporter subunit IIA	7.25E-03	2.27
BCO26_2235	enolase	1.80E-02	1.99
BCO26_2237	phosphoglycerate mutase	1.33E-02	2.08
BCO26_2238	triosephosphate isomerase	2.45E-02	1.89
BCO26_2239	phosphoglycerate kinase	1.75E-02	2.00
BCO26_2240	glyceraldehyde-3-phosphate dehydrogenase, type I	1.37E-02	2.07
BCO26_2530	fructose-bisphosphate aldolase	7.53E-04	2.83

Under the two types of lactate stress conditions, two significantly upregulated genes involved in ‘glycolysis/gluconeogenesis’ were found, namely, alcohol dehydrogenase GroES domain-containing protein (BCO26_1901) and enolase (BCO26_2235). Four key genes of glycolysis (i.e., iron-containing alcohol dehydrogenase, lactate/malate dehydrogenase, AMP-dependent synthetase and ligase and hypothetical protein BCO26_2297) were significantly down-regulated both in CA and NA compared with those in GY. Pyruvate kinase (BCO26_1967) was significantly down-regulated in NA but upregulated in CA compared with that in GY.

### Identification and comparison of DEGs involved in ‘citrate cycle (TCA cycle)’

Analysis of the genome of *B*. *coagulans* 2–6 revealed that the strain possessed all components of the TCA cycle. The TCA cycle is an important aerobic pathway in the final steps of the oxidation of carbohydrates and fatty acids. As a critical pathway for energy production in cells, it may be beneficial for *B*. *coagulans* strains to resist lactate stress conditions. Our previous study indicated that trace amounts of aeration enhance the resistance of *B*. *coagulans* to sodium lactate [5]. Therefore, we compared DEGs involved in ‘citrate cycle (TCA cycle)’ under lactate stress and non-stress conditions.

Seven significantly upregulated genes and seven significantly down-regulated genes were involved in ‘citrate cycle (TCA cycle)’ under sodium lactate stress condition (NA group) compared with those under non-stress condition (GY group) (Table 8). The pyruvate dehydrogenase complex (BCO26_0962, BCO26_0963, BCO26_0964, BCO26_0965 and BCO26_1409) was significantly upregulated as described above. Aconitate hydratase 1 (BCO26_1352), which responds to the conversion of citrate to isocitrate, and class II fumarate hydratase (BCO26_0741), which responds to the conversion of fumarate to L-malate, were also significantly upregulated. Succinyl-CoA synthetase (BCO26_1117 and BCO26_1118), 2-oxoglutarate dehydrogenase (BCO26_1275 and BCO26_1276), dihydrolipoamide dehydrogenase (BCO26_1640), succinate dehydrogenase (or fumarate reductase) cytochrome b subunit, b558 family (BCO26_1913) and isocitrate dehydrogenase (BCO26_1963) were all significantly down-regulated under sodium lactate stress.

**Table 8:** Significantly regulated genes involved in ‘citrate cycle (TCA cycle)’ under lactate stress.

Gene ID	Description	FDR	Fold change
BCO26_0741	class II fumarate hydratase	4.90E-06	3.69
BCO26_0962	pyruvate dehydrogenase E1 component subunit alpha	7.36E-05	3.15
BCO26_0963	transketolase central region	1.52E-03	2.48
BCO26_0964	hypothetical protein BCO26_0964	6.32E-03	2.13
BCO26_0965	dihydrolipoamide dehydrogenase	1.36E-02	1.92
BCO26_1352	aconitate hydratase 1	8.97E-05	3.10
BCO26_1409	dihydrolipoamide dehydrogenase	9.60E-03	2.17
BCO26_1117	succinyl-CoA synthetase subunit beta	1.58E-05	-3.51
BCO26_1118	succinyl-CoA synthetase subunit beta	1.58E-05	-3.51
BCO26_1275	succinyl-CoA synthetase subunit alpha	3.80E-04	-2.83
BCO26_1276	2-oxoglutarate dehydrogenase, E1 subunit	4.90E-02	-1.54
BCO26_1640	2-oxoglutarate dehydrogenase, E2 subunit, dihydrolipoamide succinyltransferase	4.19E-02	-1.59
BCO26_1913	dihydrolipoamide dehydrogenase	3.82E-03	-2.27
BCO26_1963	succinate dehydrogenase (or fumarate reductase) cytochrome b subunit, b558 family	1.29E-02	-2.09
BCO26_0741*	class II fumarate hydratase	2.00E-02	1.96

*Significantly regulated gene under calcium lactate stress, others are significantly regulated genes under sodium lactate stress

Only one ‘citrate cycle (TCA cycle)’ gene was found to be significantly upregulated under calcium lactate stress condition (CA group) compared with non-stress condition (GY group) (Table 8). This gene was class II fumarate hydratase (BCO26_0741), which was also expressed at higher level under sodium lactate stress compared to non-stress condition. Therefore, the TCA cycle of *B*. *coagulans* 2–6 was less affected by calcium lactate stress compared to sodium lactate stress.

### Identification of genes with at least five-fold up-regulation under lactate stress

The genes with at least five-fold up-regulation under lactate stress were identified because they are potential targets for genetic engineering to further improve *B*. *coagulans* as a producer of L-lactic acid. Under sodium lactate stress condition (NA group), 28 genes were upregulated greater than fivefold (S7 Table).

Only three genes were upregulated by greater than fivefold under calcium lactate stress condition (CA group) (S8 Table). Lambda repressor-like DNA-binding domain-containing protein (BCO26_0364) and heat shock protein Hsp20 (BCO26_1778) were both upregulated under the two types of lactate stress. The function of small heat shock proteins is to mediate correct protein folding and coping with stress-induced cell damage (Guzzo 2012). Two of the three small heat shock proteins Hsp20 in *B*. *coagulans* 2–6 were significantly upregualted under the two types of lactate stress. Major facilitator superfamily permease (BCO26_0524) was specifically upregulated greater than fivefold under calcium lactate stress (S8 Table). Five genes were specifically upregulated greater than six-fold under sodium lactate stress, namely, hypothetical protein (BCO26_0739), ATPase AAA-2 domain-containing protein (BCO26_0934), hypothetical protein (BCO26_0740), copper ion binding protein (BCO26_2102) and hypothetical protein (BCO26_2103) (S7 Table).

### Verification of RNA-seq data

Reliability of the RNA-seq data was assessed by qRT-PCR analysis. In total, 5 genes were selected for validation. Although the magnitude of fold-change in gene expression varied between the two data sets, similar trends were observed in qRT-PCR and RNA-seq results, which supported the validity of the RNA-seq data (S9 Table).

## Discussion

*B*. *coagulans* strains have attracted significant attention for L-lactic acid production in the past few years [5, 11, 25–28]. Most of the research on this strain has focused on fermentation process. Qin et al. [11] and Ye et al. [28] reported that Ca(OH)_2_ and CaCO_3_ are more suitable neutralizing agents than NaOH for getting higher lactic acid titer. They presumed that it was attributable to lower product inhibition by calcium lactate compared to sodium lactate. However, little is known about the molecular mechanisms by which *B*. *coagulans* responds to different lactates.

In this study, the gene expression patterns of *B*. *coagulans* 2–6 responding to sodium lactate and calcium lactate were investigated by RNA-seq and comparative transcriptome analysis. Gene ontology assignments and KEGG pathway enrichment analysis of the DEGs were performed. It was found that the influence of sodium lactate on gene expression in *B*. *coagulans* 2–6 was greater than that of calcium lactate. Sodium lactate induced significant upregulation of 543 genes and down-regulation of 670 genes, while calcium lactate induced significant upregulation of 398 genes and down-regulation of 314 genes. It was also found that lactate fermentation was less affected by calcium lactate stress than by sodium lactate stress. Key genes of the glycolysis pathway were most significantly upregulated under calcium lactate stress, while L-lactate dehydrogenase (–1.52-fold), pyruvate kinase (–3.98-fold) and 6-phosphofructokinase (–1.41-fold) were down-regulated under sodium lactate stress. Pyruvate dehydrogenase complex, which converts pyruvic acid to acetyl-CoA, was significantly upregulated (2.8-fold) under sodium lactate stress. These changes are expected to have significant influence on the formation of lactic acid from pyruvic acid. Taken together, our findings provide new mechanistic insights into higher lactic acid titer obtained using CaCO_3_ as neutralizing agent compared to that obtained using NaOH [5, 11].

Our results also provide potential targets for genetic engineering to further improve *B*. *coagulans* as an L-lactic acid producer. ABC transporters play important roles in response to lactate stress. A large number of the ABC transporters in *B*. *coagulans* 2–6 were found to be significantly down-regulated under the two types of lactate stress. We also identified ABC transporters that were significantly upregulated under the two types of lactates stress or specifically upregulated by one lactate. High expression of ABC transporter genes may be of benefit to *B*. *coagulans* 2–6 to maintain intercellular homeostasis; as such, these genes are good potential targets for genetic engineering. Furthermore, we identified genes with at least five-fold upregulation under lactate stress. Some of them were well-known stress-induced proteins, such as heat shock protein Hsp20 [29] and major facilitator superfamily protein [30], while others were previously unknown proteins, whose functions could be characterized in future studies.

Wang et al. [12] carried out a comparative proteomic analysis of *B*. *coagulans* in response to lactate stress. Using two-dimensional electrophoresis coupled with mass spectrometric identification, they identified four highly expressed proteins under calcium lactate stress. The same four proteins were also identified in this study, which indicates that our results are consistent with previous findings on this topic. In addition, we found that expressions of lactate dehydrogenase (ACR02673), cysteine synthase A (AEH52120) and ribosomal protein L7/L12 (AEH52167) were upregulated under calcium lactate stress but down-regulated under sodium lactate stress. Gene expression of aldo/keto reductase (AEH52329) was upregulated under calcium lactate stress and sodium lactate stress. Thus, our RNA-seq dataset provides a rich account of gene expression changes in *B*. *coagulans* in response to lactate stress, which could be highly useful for future efforts to improve lactic acid production by genetic engineering.

## Conclusions

In summary, RNA-seq technology was employed in this study to characterise the transcriptome of *B*. *coagulans* under non-stress, sodium lactate stress and calcium lactate stress conditions. The responses of *B*. *coagulans* 2–6 to sodium lactate stress include: more genes changed expression than that in response to calcium lactate stress, significant regulation of ‘amino sugar and nucleotide sugar metabolism’ genes and negative influence on ‘glycolysis/gluconeogenesis’ and ‘citrate cycle (TCA cycle)’ genes. The responses of *B*. *coagulans* 2–6 to calcium lactate stress include: less genes changed expression than that in response to sodium lactate stress, significant regulation of ‘ABC transporters’ genes and positive influence on ‘glycolysis/gluconeogenesis’ genes. These results provide new insights into the responses of *B*. *coagulans* to different lactate stresses. Notably, our findings not only provide a robust database for investigating the functions of genes induced by lactate stress but also identify potential targets for genetic engineering to further improve L-lactic acid production by *B*. *coagulans*.

## Supporting Information

S1 Table The primers for qRT-PCR analysis with target gene information. (DOC)

S2 Table Significantly down-regulated genes involved in ‘ABC transporters’ under calcium lactate stress. (DOC)

S3 Table Significantly down-regulated genes involved in ‘ABC transporters’ under sodium lactate stress. (DOC)

S4 Table Significantly down-regulated genes involved in ‘amino sugar and nucleotide sugar metabolism’ under sodium lactate stress. (DOC)

S5 Table Significantly down-regulated genes involved in ‘glycolysis/gluconeogenesis’ under sodium lactate stress. (DOC)

S6 Table Significantly down-regulated genes involved in ‘glycolysis/gluconeogenesis’ under calcium lactate stress. (DOC)

S7 Table Genes with at least fivefold upregulation under sodium lactate stress. (DOC)

S8 Table Genes with at least fivefold upregulation under calcium lactate stress. (DOC)

S9 Table The expression information of selected genes for qRT-PCR under lactate stress condition. (DOC)

S1 Data GO assignments of differentially expressed genes (CA vs GY). (XLS)

S2 Data GO assignments of differentially expressed genes (NA vs GY). (XLS)
