# Supplementary material for: Comparative Transcriptome Analysis Reveals Different Molecular Mechanisms of Bacillus coagulans 2-6 Response to Sodium Lactate and Calcium Lactate during Lactic Acid Production
Source: PLoS One. 2015 Apr 15;10(4):e0124316. doi: 10.1371/journal.pone.0124316 (PMC4398400; doi:10.1371/journal.pone.0124316)
Supplement: S1 Table — (DOC) [file pone.0124316.s001.doc]

**Table S1.** The primers for qRT-PCR analysis with target gene information

| **Gene ID** | **Description** | **Primers (5΄→3΄)** | **Length** |
| --- | --- | --- | --- |
| BCO26_0061 | cysteine synthase A | CGGAACAACTCGTGAAAGA  GGTTGTCAAACGGTGGAT | 86 bp |
| BCO26_0531 | L-lactate dehydrogenase | GCAAAAGCAGAAGGGGAAGC  GCAGTGCCGCAATCGGAATA | 101 bp |
| BCO26_0934 | ATPase AAA-2 domain-containing protein | GGATTCGGAGGATTCAACG  CGGCCAAACTGGTCAAGTAG | 128 bp |
| BCO26_1967 | pyruvate kinase | ACGGTTTGATTGAACTGGAA  AATGAAGCCGCAATGAAGT | 205 bp |
| BCO26_2724 | chaperonin GroEL | CGAACGATGTAGCTGGTG  AGCAACTTGGGCGATGGA | 203 bp |
| BCO26_R00002 | 16S rRNA | AGGCTGAAACTCAAAGGAAT  AACCCAACATCTCACGACAC | 193 bp |
