# Supplementary material for: Comparative Transcriptome Analysis Reveals Different Molecular Mechanisms of Bacillus coagulans 2-6 Response to Sodium Lactate and Calcium Lactate during Lactic Acid Production
Source: PLoS One. 2015 Apr 15;10(4):e0124316. doi: 10.1371/journal.pone.0124316 (PMC4398400; doi:10.1371/journal.pone.0124316)
Supplement: S2 Table — (DOC) [file pone.0124316.s002.doc]

**Table S2. Significantly down-regulated genes involved in ‘ABC transporters’ under calcium lactate stress**

| **Gene ID** | **Description** | **FDR** | **Fold change** |
| --- | --- | --- | --- |
| BCO26_0232 | periplasmic binding protein | 4.08E-03 | -2.43 |
| BCO26_0276 | ABC transporter-like protein | 1.91E-04 | -3.19 |
| BCO26_0277 | spermidine/putrescine ABC transporter membrane protein | 1.43E-04 | -3.31 |
| BCO26_0278 | binding-protein-dependent transport system inner membrane protein | 6.81E-04 | -2.98 |
| BCO26_0279 | family 1 extracellular solute-binding protein | 9.92E-04 | -2.85 |
| BCO26_0315 | ABC transporter-like protein | 3.75E-04 | -3.30 |
| BCO26_0317 | family 3 extracellular solute-binding protein | 5.85E-04 | -3.03 |
| BCO26_0318 | polar amino acid ABC transporter inner membrane subunit | 2.74E-02 | -2.24 |
| BCO26_0450 | cobalt transport protein | 2.62E-06 | -5.17 |
| BCO26_0451 | sigma 54 interacting domain-containing protein | 1.51E-05 | -3.99 |
| BCO26_0466 | phosphonate ABC transporter permease | 4.65E-02 | -1.70 |
| BCO26_0467 | phosphonate ABC transporter inner membrane subunit | 3.90E-03 | -2.47 |
| BCO26_0468 | phosphonate ABC transporter phosphonate-binding protein | 1.90E-02 | -1.99 |
| BCO26_0676 | cell division ATP-binding protein FtsE | 1.48E-02 | -2.06 |
| BCO26_0677 | hypothetical protein BCO26_0677 | 3.56E-03 | -2.45 |
| BCO26_0828 | binding-protein-dependent transport system inner membrane protein | 2.80E-04 | -3.11 |
| BCO26_0829 | binding-protein-dependent transport system inner membrane protein | 1.55E-03 | -2.70 |
| BCO26_0830 | oligopeptide/dipeptide ABC transporter ATPase | 1.30E-03 | -2.73 |
| BCO26_0831 | ABC transporter-like protein | 1.20E-02 | -2.14 |
| BCO26_2255 | family 5 extracellular solute-binding protein | 1.19E-04 | -3.48 |
| BCO26_2256 | oligopeptide/dipeptide ABC transporter ATPase | 1.95E-05 | -4.61 |
| BCO26_2257 | binding-protein-dependent transport system inner membrane protein | 1.24E-04 | -4.30 |
| BCO26_2258 | binding-protein-dependent transport system inner membrane protein | 3.42E-02 | -2.04 |
| BCO26_2763 | family 1 extracellular solute-binding protein | 2.90E-03 | -2.51 |
| BCO26_2764 | binding-protein-dependent transport system inner membrane protein | 1.41E-07 | -4.97 |
| BCO26_2765 | binding-protein-dependent transport system inner membrane protein | 2.95E-05 | -3.79 |
| BCO26_2892 | ABC transporter-like protein | 2.80E-04 | -3.10 |
