# Supplementary material for: Comparative Transcriptome Analysis Reveals Different Molecular Mechanisms of Bacillus coagulans 2-6 Response to Sodium Lactate and Calcium Lactate during Lactic Acid Production
Source: PLoS One. 2015 Apr 15;10(4):e0124316. doi: 10.1371/journal.pone.0124316 (PMC4398400; doi:10.1371/journal.pone.0124316)
Supplement: S3 Table — (DOC) [file pone.0124316.s003.doc]

**Table S3. Significantly down-regulated genes involved in ‘ABC transporters’ under sodium lactate stress**

| **Gene ID** | **Description** | **FDR** | **Fold change** |
| --- | --- | --- | --- |
| BCO26_0202 | glycine betaine/L-proline ABC transporter ATPase | 3.74E-03 | -2.35 |
| BCO26_0203 | glycine betaine ABC transporter substrate-binding protein | 6.17E-03 | -2.20 |
| BCO26_0232 | periplasmic binding protein | 6.16E-06 | -3.76 |
| BCO26_0276 | ABC transporter-like protein | 7.08E-06 | -3.70 |
| BCO26_0277 | spermidine/putrescine ABC transporter membrane protein | 2.96E-06 | -4.00 |
| BCO26_0278 | binding-protein-dependent transport system inner membrane protein | 7.36E-04 | -2.76 |
| BCO26_0279 | family 1 extracellular solute-binding protein | 2.62E-03 | -2.41 |
| BCO26_0315 | ABC transporter-like protein | 7.87E-07 | -4.88 |
| BCO26_0317 | family 3 extracellular solute-binding protein | 3.29E-04 | -2.98 |
| BCO26_0318 | polar amino acid ABC transporter inner membrane subunit | 2.59E-02 | -2.08 |
| BCO26_0450 | cobalt transport protein | 1.31E-08 | -9.39 |
| BCO26_0451 | sigma 54 interacting domain-containing protein | 1.36E-08 | -5.65 |
| BCO26_0465 | phosphonate ABC transporter ATPase | 6.97E-05 | -3.28 |
| BCO26_0466 | phosphonate ABC transporter permease | 1.20E-06 | -4.11 |
| BCO26_0467 | phosphonate ABC transporter inner membrane subunit | 7.95E-05 | -3.25 |
| BCO26_0468 | phosphonate ABC transporter phosphonate-binding protein | 2.13E-05 | -3.46 |
| BCO26_0676 | cell division ATP-binding protein FtsE | 7.08E-06 | -3.64 |
| BCO26_0677 | hypothetical protein BCO26_0677 | 1.44E-06 | -3.97 |
| BCO26_0709 | phosphate ABC transporter ATPase | 1.23E-02 | -2.80 |
| BCO26_0827 | family 5 extracellular solute-binding protein | 1.16E-09 | -5.29 |
| BCO26_0828 | binding-protein-dependent transport system inner membrane protein | 9.76E-10 | -5.66 |
| BCO26_0829 | binding-protein-dependent transport system inner membrane protein | 2.35E-08 | -4.94 |
| BCO26_0830 | oligopeptide/dipeptide ABC transporter ATPase | 8.07E-07 | -4.14 |
| BCO26_0831 | ABC transporter-like protein | 1.68E-05 | -3.53 |
| BCO26_1441 | hypothetical protein BCO26_1441 | 4.62E-06 | -3.82 |
| BCO26_1442 | ABC transporter-like protein | 7.18E-05 | -3.23 |
| BCO26_2019 | ABC transporter-like protein | 3.00E-02 | -1.75 |
| BCO26_2256 | oligopeptide/dipeptide ABC transporter ATPase | 9.63E-03 | -2.24 |
| BCO26_2258 | binding-protein-dependent transport system inner membrane protein | 1.11E-03 | -3.03 |
| BCO26_2763 | family 1 extracellular solute-binding protein | 3.22E-05 | -3.36 |
| BCO26_2764 | binding-protein-dependent transport system inner membrane protein | 1.76E-07 | -4.64 |
| BCO26_2765 | binding-protein-dependent transport system inner membrane protein | 2.20E-08 | -5.41 |
| BCO26_2890 | binding-protein-dependent transport system inner membrane protein | 6.63E-03 | -2.19 |
| BCO26_2891 | ABC transporter-like protein | 1.74E-02 | -1.87 |
| BCO26_2892 | ABC transporter-like protein | 1.58E-07 | -4.48 |
