# Supplementary material for: Comparative Transcriptome Analysis Reveals Different Molecular Mechanisms of Bacillus coagulans 2-6 Response to Sodium Lactate and Calcium Lactate during Lactic Acid Production
Source: PLoS One. 2015 Apr 15;10(4):e0124316. doi: 10.1371/journal.pone.0124316 (PMC4398400; doi:10.1371/journal.pone.0124316)
Supplement: S4 Table — (DOC) [file pone.0124316.s004.doc]

**Table S4. Significantly down-regulated genes involved in ‘amino sugar and nucleotide sugar metabolism’ under sodium lactate stress**

| **Gene ID** | **Description** | **FDR** | **Fold change** |
| --- | --- | --- | --- |
| BCO26_0341 | UTP-glucose-1-phosphate uridylyltransferase | 4.51E-03 | -2.24 |
| BCO26_0343 | NAD-dependent epimerase/dehydratase | 3.31E-08 | -5.03 |
| BCO26_0354 | nucleotide sugar dehydrogenase | 1.11E-02 | -1.98 |
| BCO26_0540 | PTS system mannose/fructose/sorbose family transporter subunit IIB | 1.46E-03 | -2.54 |
| BCO26_0541 | PTS system mannose/fructose/sorbose family transporter subunit IIA | 9.12E-06 | -3.59 |
| BCO26_0542 | PTS system sorbose-specific transporter subunit IIC | 2.09E-05 | -3.45 |
| BCO26_0543 | PTS system mannose/fructose/sorbose family transporter subunit IID | 9.12E-06 | -3.59 |
| BCO26_0632 | mannose-6-phosphate isomerase | 7.09E-03 | -2.12 |
| BCO26_0869 | ROK family protein | 1.92E-02 | -1.90 |
| BCO26_2322 | RpiR family transcriptional regulator | 3.13E-02 | -1.98 |
| BCO26_2790 | UDP-N-acetylglucosamine 1-carboxyvinyltransferase | 1.23E-03 | -2.53 |
