# Supplementary material for: Comparative Transcriptome Analysis Reveals Different Molecular Mechanisms of Bacillus coagulans 2-6 Response to Sodium Lactate and Calcium Lactate during Lactic Acid Production
Source: PLoS One. 2015 Apr 15;10(4):e0124316. doi: 10.1371/journal.pone.0124316 (PMC4398400; doi:10.1371/journal.pone.0124316)
Supplement: S5 Table — (DOC) [file pone.0124316.s005.doc]

**Table S5. Significantly down-regulated genes involved in ‘glycolysis/gluconeogenesis’ under sodium lactate stress**

| **Gene ID** | **Description** | **FDR** | **Fold change** |
| --- | --- | --- | --- |
| BCO26_0567 | iron-containing alcohol dehydrogenase | 1.57E-06 | -3.93 |
| BCO26_1424 | lactate/malate dehydrogenase | 6.07E-07 | -4.22 |
| BCO26_1640 | dihydrolipoamide dehydrogenase | 3.82E-03 | -2.27 |
| BCO26_1967 | pyruvate kinase | 5.05E-06 | -3.98 |
| BCO26_1992 | AMP-dependent synthetase and ligase | 1.04E-03 | -2.82 |
| BCO26_2022 | AMP-dependent synthetase and ligase | 4.98E-02 | -1.55 |
| BCO26_2297 | hypothetical protein BCO26_2297 | 2.47E-03 | -2.50 |
| BCO26_2625 | alcohol dehydrogenase GroES domain-containing protein | 4.04E-02 | -1.90 |
