# Supplementary material for: Comparative Transcriptome Analysis Reveals Different Molecular Mechanisms of Bacillus coagulans 2-6 Response to Sodium Lactate and Calcium Lactate during Lactic Acid Production
Source: PLoS One. 2015 Apr 15;10(4):e0124316. doi: 10.1371/journal.pone.0124316 (PMC4398400; doi:10.1371/journal.pone.0124316)
Supplement: S6 Table — (DOC) [file pone.0124316.s006.doc]

**Table S6.** Significantly down-regulated genes involved in ‘glycolysis/gluconeogenesis’ under calcium lactate stress

| **Gene ID** | **Description** | **FDR** | **Fold change** |
| --- | --- | --- | --- |
| BCO26_0567 | iron-containing alcohol dehydrogenase | 4.23E-04 | -2.97 |
| BCO26_1424 | lactate/malate dehydrogenase | 2.88E-03 | -2.53 |
| BCO26_2022 | AMP-dependent synthetase and ligase | 4.74E-02 | -1.70 |
| BCO26_2141 | aldehyde dehydrogenase | 4.93E-02 | -1.95 |
| BCO26_2297 | hypothetical protein BCO26_2297 | 8.13E-03 | -2.32 |
