# Supplementary material for: Comparative Transcriptome Analysis Reveals Different Molecular Mechanisms of Bacillus coagulans 2-6 Response to Sodium Lactate and Calcium Lactate during Lactic Acid Production
Source: PLoS One. 2015 Apr 15;10(4):e0124316. doi: 10.1371/journal.pone.0124316 (PMC4398400; doi:10.1371/journal.pone.0124316)
Supplement: S7 Table — (DOC) [file pone.0124316.s007.doc]

**Table S7.** Genes with at least fivefold upregulation under sodium lactate stress

| **Gene ID** | **Description** | **FDR** | **Fold change** |
| --- | --- | --- | --- |
| BCO26_0739 | hypothetical protein BCO26_0739 | 1.40E-13 | 7.03 |
| BCO26_1778 | heat shock protein Hsp20 | 7.89E-14 | 7.02 |
| BCO26_0934 | ATPase AAA-2 domain-containing protein | 1.40E-13 | 6.89 |
| BCO26_0740 | hypothetical protein BCO26_0740 | 9.10E-13 | 6.77 |
| BCO26_2102 | copper ion binding protein | 1.13E-12 | 6.53 |
| BCO26_2103 | hypothetical protein BCO26_2103 | 4.98E-12 | 6.21 |
| BCO26_0364 | lambda repressor-like DNA-binding domain-containing protein | 1.07E-10 | 5.83 |
| BCO26_2192 | FeS assembly ATPase SufC | 1.88E-10 | 5.60 |
| BCO26_0858 | hypothetical protein BCO26_0858 | 3.09E-10 | 5.59 |
| BCO26_2462 | isopentenyl-diphosphate delta-isomerase | 5.74E-10 | 5.42 |
| BCO26_0365 | hypothetical protein BCO26_0365 | 1.00E-09 | 5.38 |
| BCO26_2159 | major facilitator superfamily protein | 9.32E-10 | 5.37 |
| BCO26_0775 | antibiotic biosynthesis monooxygenase | 7.65E-10 | 5.37 |
| BCO26_0158 | KinB signaling pathway activation protein | 7.54E-05 | 5.36 |
| BCO26_2101 | heavy metal translocating P-type ATPase | 9.32E-10 | 5.32 |
| BCO26_1259 | transcriptional regulator TrmB | 1.16E-09 | 5.31 |
| BCO26_1902 | Coat F domain-containing protein | 1.14E-07 | 5.28 |
| BCO26_1539 | peptidase membrane zinc metallopeptidase putative | 1.16E-09 | 5.28 |
| BCO26_2902 | thiamine pyrophosphate domain-containing TPP-binding protein | 1.16E-09 | 5.26 |
| BCO26_0255 | glucosamine/fructose-6-phosphate aminotransferase | 2.00E-09 | 5.17 |
| BCO26_1901 | alcohol dehydrogenase GroES domain-containing protein | 4.14E-09 | 5.13 |
| BCO26_2463 | N-acylglucosamine-6-phosphate 2-epimerase | 3.29E-09 | 5.08 |
| BCO26_1731 | hypothetical protein BCO26_1731 | 1.90E-08 | 5.06 |
| BCO26_0815 | hypothetical protein BCO26_0815 | 2.20E-06 | 5.05 |
| BCO26_0213 | flavodoxin/nitric oxide synthase | 4.79E-09 | 5.05 |
| BCO26_0427 | transferase hexapeptide repeat containing protein | 5.08E-09 | 5.01 |
| BCO26_2948 | hypothetical protein BCO26_2948 | 4.66E-06 | 5.01 |
| BCO26_1187 | YlmC/YmxH family sporulation protein | 5.74E-09 | 5.00 |
