# Supplementary material for: Comparative Transcriptome Analysis Reveals Different Molecular Mechanisms of Bacillus coagulans 2-6 Response to Sodium Lactate and Calcium Lactate during Lactic Acid Production
Source: PLoS One. 2015 Apr 15;10(4):e0124316. doi: 10.1371/journal.pone.0124316 (PMC4398400; doi:10.1371/journal.pone.0124316)
Supplement: S8 Table — (DOC) [file pone.0124316.s008.doc]

**Table S8. Genes with at least fivefold upregulation under calcium lactate stress**

| **Gene ID** | **Description** | **FDR** | **Fold change** |
| --- | --- | --- | --- |
| BCO26_0364 | lambda repressor-like DNA-binding domain-containing protein | 3.61E-10 | 5.99 |
| BCO26_0524 | major facilitator superfamily permease | 3.79E-09 | 5.47 |
| BCO26_1778 | heat shock protein Hsp20 | 2.42E-09 | 5.46 |
