# Supplementary material for: Comparative Transcriptome Analysis Reveals Different Molecular Mechanisms of Bacillus coagulans 2-6 Response to Sodium Lactate and Calcium Lactate during Lactic Acid Production
Source: PLoS One. 2015 Apr 15;10(4):e0124316. doi: 10.1371/journal.pone.0124316 (PMC4398400; doi:10.1371/journal.pone.0124316)
Supplement: S9 Table — (DOC) [file pone.0124316.s009.doc]

**Table S9.** The expression information of selected genes for qRT-PCR under lactate stress condition

| **Gene ID** | **Description** | Fold change (CA vs GY) | | Fold change(NA vs GY) | |
| --- | --- | --- | --- | --- | --- |
|  |  | RNA-seq | qRT-PCR | RNA-seq | qRT-PCR |
| BCO26_0061 | cysteine synthase A | 1.79 | 1.65 | -0.37 | -2.94 |
| BCO26_0531 | L-lactate dehydrogenase | 3.22 | 2.08 | -1.51 | -1.96 |
| BCO26_0934 | ATPase AAA-2 domain-containing protein | 4.48 | 4.77 | 6.89 | 4.67 |
| BCO26_1967 | pyruvate kinase | 2.33 | 1.45 | -1.88 | -2.78 |
| BCO26_2724 | chaperonin GroEL | 4.48 | 2.8 | 2.23 | 1.51 |
